# Thromboelastometry-guided haemostatic resuscitation in severely injured patients: a propensity score-matched study

**DOI:** 10.1186/s13054-023-04421-w

**Published:** 2023-04-13

**Authors:** Jean-Stéphane David, Arthur James, Maxime Orion, Agathe Selves, Mélody Bonnet, Pauline Glasman, Charles-Hervé Vacheron, Mathieu Raux

**Affiliations:** 1 Department of Anesthesia and Intensive Care, Groupe Hospitalier Sud, Hospices Civils de Lyon (HCL), Pierre Bénite Cedex, France; 2 Research on Healthcare Performance (RESHAPE), INSERM U1290, University Claude Bernard Lyon 1, Lyon, France; 3 GRC 29, AP-HP, DMU DREAM, Department of Anaesthesia and Intensive Care, Pitié-Salpêtrière Hospital, Sorbonne University, Paris, France; 4 Biometrics and Evolutionary Biology Laboratory, Biostatistics-Health Team, HCL, Villeurbanne, France; 5 Division of Public Health, Department of Biostatistics and Bioinformatics, Lyon, France; 6 INSERM, UMRS1158 Neurophysiologie Respiratoire Expérimentale Et Clinique; AP-HP, Groupe Hospitalier Universitaire APHP-Sorbonne Université, Site Pitié-Salpêtrière, Département d’Anesthésie Réanimation, Sorbonne Université, Paris, France

**Keywords:** Thromboelastometry, Blood products, Trauma, Cost, Outcome

## Abstract

**Background:**

To accelerate the diagnosis and treatment of trauma-induced coagulopathy (TIC), viscoelastic haemostatic assays (VHA) are increasingly used worldwide, although their value is still debated, with a recent randomised trial showing no improvement in outcome. The objective of this retrospective study was to compare 2 cohorts of injured patients in which TIC was managed with either a VHA-based algorithm or a conventional coagulation test (CCT)-based algorithm.

**Methods:**

Data were retrieved from 2 registries and patients were included in the study if they received at least 1 unit of red blood cell in the first 24 h after admission. A propensity score, including sex, age, blunt vs. penetrating, systolic blood pressure, GCS, ISS and head AIS, admission lactate and PT_ratio_, tranexamic acid administration, was then constructed. Primary outcome was the proportion of subjects who were alive and free of massive transfusion (MT) at 24 h after injury. We also compared the cost for blood products and coagulation factors.

**Results:**

From 2012 to 2019, 7250 patients were admitted in the 2 trauma centres, and among these 624 were included in the study (CCT group: 380; VHA group: 244). After propensity score matching, 215 patients remained in each study group without any significant difference in demographics, vital signs, injury severity, or laboratory analysis. At 24 h, more patients were alive and free of MT in the VHA group (162 patients, 75%) as compared to the CCT group (112 patients, 52%; *p* < 0.01) and fewer patients received MT (32 patients, 15% vs. 91 patients, 42%, *p* < 0.01). However, no significant difference was observed for mortality at 24 h (odds ratio 0.94, 95% CI 0.59–1.51) or survival at day 28 (odds ratio 0.87, 95% CI 0.58–1.29). Overall cost of blood products and coagulation factors was dramatically reduced in the VHA group as compared to the CCT group (median [interquartile range]: 2357 euros [1108–5020] vs. 4092 euros [2510–5916], *p* < 0.001).

**Conclusions:**

A VHA-based strategy was associated with an increase of the number of patients alive and free of MT at 24 h together with an important reduction of blood product use and associated costs. However, that did not translate into an improvement in mortality.

**Supplementary Information:**

The online version contains supplementary material available at 10.1186/s13054-023-04421-w.

## Introduction

Severe injury remains the leading cause of death before 45 years of age in developed countries [1]. Early death (< 24 h) is dominated by haemorrhage, which represents also the first cause of avoidable death [2, 3]. Trauma-induced coagulopathy (TIC) is a specific haemostasis entity seen in approximately 25% of patients [4], and is directly associated with blood product transfusion requirement, organ failure, and death [4, 5]. It has a complex physiopathology that includes various phenomena such as the loss and consumption of coagulation factors, fibrinolysis activation, and dysfunction of platelets and the endothelium [4]. TIC is further exacerbated by shock and hypoperfusion, resuscitation with large volumes of crystalloids, but also hypothermia, acidosis, and hypocalcaemia [4]. The management of TIC is therefore a major challenge during the first hours following injury since it has been shown that early diagnosis and correction of TIC is associated with improved outcome [5, 6]. In cases of uncontrolled haemorrhage, the initial strategy is to stop the haemorrhage by surgery and interventional radiology in combination with blood resuscitation including the administration of predefined ratios of blood products (BP) [5, 7]. This haemostatic resuscitation leads to improved outcomes but often fails to correct the coagulopathy and may cause overconsumption of BP [8, 9].

It is nowadays suggested to use viscoelastic haemostatic assay (VHA) rather than conventional coagulation tests (CCT) to prompt diagnosis of TIC and to guide the administration of BP [5, 10]. However, while using VHAs to reduce BP administration and morbidity has well-established benefits in various settings including cardiac surgery [11, 12] and liver transplantation [13], questions persist in injured patients [14] and conflicting results have been published. Several retrospective studies have shown an important reduction in BP administration and even an improvement in outcome [15–18]. However, in the studies by Stein and Guth, it was not possible to attribute the positive results to the implementation of a TVE alone, but rather to the implementation of a bundle of care including TVEs, the administration of tranexamic acid and the use of *Damage Control* strategies. More recently, a randomised trial showed no reduction in BP administration and no improvement in outcomes, but it should be noted that less than 25% of patients had a PT_ratio_ > 1.20, which decreased the likelihood of demonstrating a difference between study groups [19, 20].

It is in response to these criticisms that we propose to evaluate the impact of a VHA-based versus a CCT-based TIC management strategy on BP administration and the outcomes, in a population of severely injured patients with a high probability of TIC, who otherwise, all benefit from tranexamic acid administration and damage control strategies. Our hypothesis was that the use of a VHA-based algorithm in a patient population with a high probability of TIC was associated with improved outcomes.

## Methodology

### Study design

This was a retrospective analysis of data from patients who sustained a severe injury, admitted from 1 January 2012 31 December 2019 to the trauma resuscitation unit of two level-I trauma centres located in major university hospitals (CCT group: Groupe Hospitalier Pitié-Salpétrière, Paris; VHA group: Groupe Hospitalier Lyon Sud, Pierre-Benite). The data were retrieved from two prospectively populated registries in which data were collected (TraumaBase, https://www.traumabase.eu/fr; and RESUVAL, https://www.resuval.com).

The regional emergency network RESUVAL supervised the registry and obtained approval from the national data protection commission (CNIL: *Commission Nationale Informatique et Liberte*, DE 2012–059), the national committee for data protection in medical research (*Comité consultatif sur le traitement de l'information en matière de recherche*, CCTIRS), and the institutional review board (02/2020). The TraumaBase database was approved by the CCTIRS (CCTIRS 11.305 bis) and the CNIL (CNIL 911,461). In both registries, prehospital and in-hospital data are recorded. Written informed consent was not required, and all patients (or their next of kin) were provided with information about the registry. This article adheres to the *Strengthening the Reporting of Observational Studies in Epidemiology* (STROBE) guidelines.

### Patient care

*Apart from the management of haemostasis, which was done according to an algorithm based on the CCT at the Pitié-Salpétrière hospital and according to an algorithm based on the VHA at the Lyon-Sud hospital, the management of patients was organised in the same way.* As it is usual practice in France, before to be admitted in the trauma resuscitation unit, all patients were cared for and triaged during the prehospital phase by a physician who may be an anaesthesiologist-intensivist or an emergency medicine physician (‘SAMU system’) [21]. During prehospital care, doctors assess the severity of the trauma (clinical examination, vital signs, FAST examination and point-of-care haemoglobin) and direct the patient to the most appropriate facility. At the same time, they will implement all the necessary care according to the injury and vital distress, including all the relevant resuscitation techniques (mechanical ventilation, blood transfusion, vasopressor and vascular filling, analgesia, chest tube, etc.) [22]. In case of cardiac arrest, standard CPR was initiated in association with life-saving measures including bilateral chest decompression, external haemorrhage control, airway control, transfusion or fluid resuscitation [23].

In both hospitals, the TRU is located in a post-anaesthesia care unit that is in close proximity to the emergency surgery rooms. The TRU and the emergency surgery rooms are supervised by an anaesthesiologist-intensivist who also acts as the "Trauma Team Leader". In-hospital care included the use of damage control resuscitation strategies [24]. Trauma surgeons are present in the TRU at admission of patients in shock and interventional radiology is available 24 h a day.

### Patient selection

To ensure that patients were sufficiently injured and had increased probability of TIC, we included only patients that receive at least 1 red blood cell (RBC) unit in the first 24 h, including prehospital transfusion [25]. Patients < 16 years, receiving anticoagulant therapy, or transferred from another hospital were excluded. As well, we excluded in the VHA group, 17 patients who did not have a thromboelastometry analysis (ROTEM Delta, Werfen, Le Pré Saint Gervais, France) during care.

For each patient, we recorded the demographic and injury characteristics including the injury severity score (ISS), intensive care unit (ICU) length of stay, survival at 24 h and day 28, and haemorrhage control procedures done in the first 6-h following the admission (laparotomy or pelvic packing, thoracotomy, interventional radiology). Laboratory analyses at admission were also retrieved from the registries including CCT and VHA results. TIC was defined by a PT_ratio_ at admission > 1.2 or, in the absence of conventional coagulation results, a A5 EXTEM < 36 mm [26].

### Blood product administration

During the first 24 h, all BP given to the patients were registered. Treatment options for patients with coagulopathy included fibrinogen concentrates (FC; Clottafact^®^, LFB, Les Ullis, France) for a fibrinogen deficit, fresh frozen plasma (FFP), and/or prothrombin complex concentrate (PCC: Kanokad^®^ (LFB, Les Ullis, France) or Octaplex^®^ (Octapharma, Boulogne Billancourt, France)) for a coagulation factor deficit, and platelet concentrate (PC) for thrombocytopenia.

**Fig. 1:**
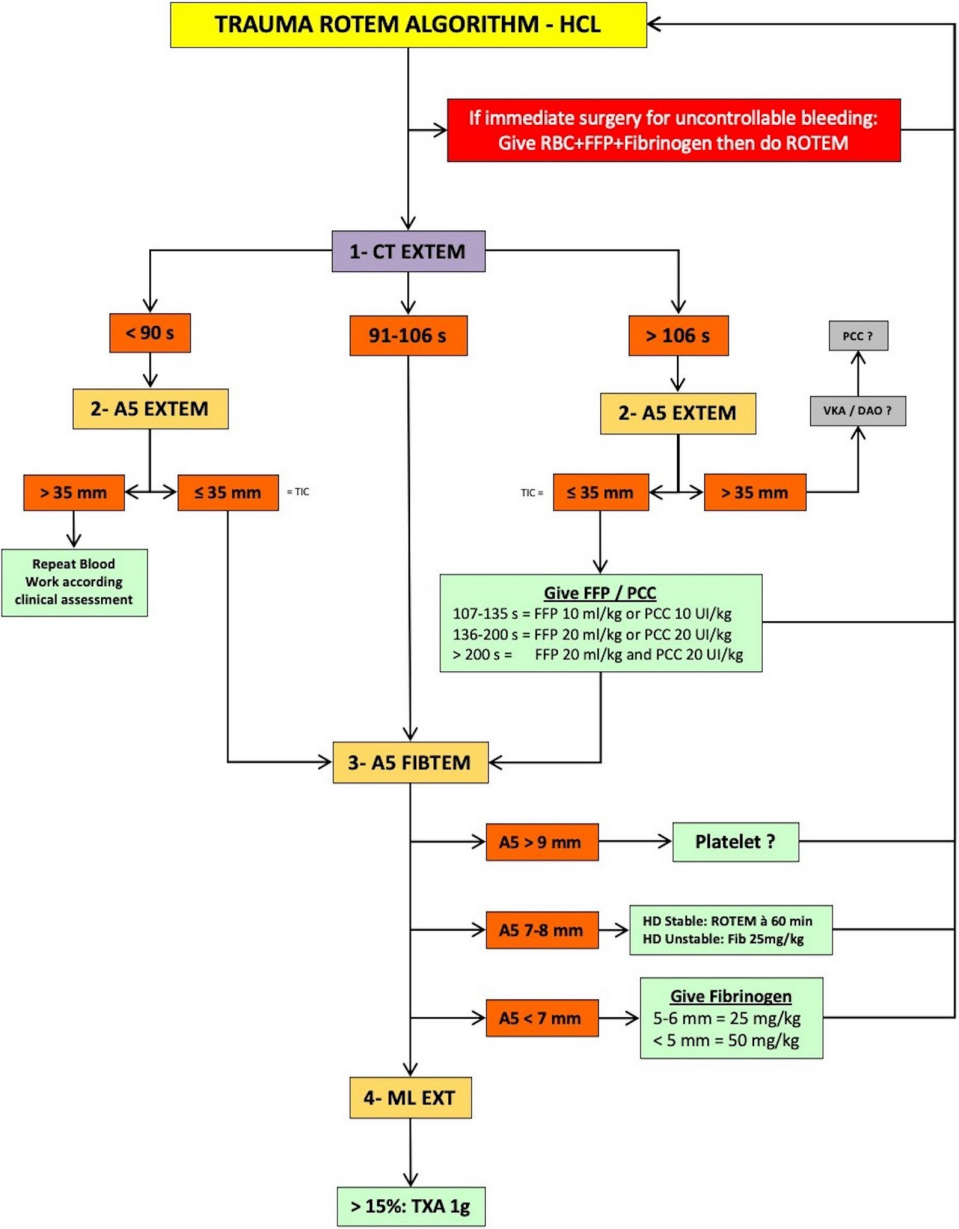
Algorithm used in the VHA group

In the VHA group, administration of haemostatic products was guided by thromboelastometry according to clinical judgement and an algorithm locally developed (Fig. 1). ROTEM analysis was done at admission or during follow-up because of clinical impairment or at the discretion of the attending physician. The ROTEM analysis was performed at 37 °C, in parallel, on two channels (EXTEM and FIBTEM). The following ROTEM parameters were analysed: clotting time (CT) and the amplitude of clot at 5 min (A5) [27]. ROTEM analyses were performed in a standardised fashion throughout the study period in the haemostasis laboratory where the ROTEM is located. The results were immediately available on the computer located in the trauma resuscitation unit or in the operating room.

In the CCT group, BP were given based on conventional coagulation tests and clinical judgment with the goal of achieving a FFP/RBC/platelet ratio close to 1. While the treatment decision was ultimately at the discretion of the attending physician, both French and European guidelines were used [5, 28].

Fibrinogen concentrate was given if the fibrinogen was < 1.5 g/L or if there was evidence of fibrinogen deficit on the ROTEM analysis (A5 Fibtem < 7 mm) [5, 17]. FFPs were given in the CCT group if the PT was < 40% or in combination with RBC to achieve a FFP/RBC ratio of 1:1. FFPs and/or PCCs were given in the VHA group, if there was evidence of coagulation factors deficit (EXTEM CT > 90 s and FIBTEM A5 > 6 mm or EXTEM CT > 106 s) [17]. In the VHA group, administration of FFP and fibrinogen was possible before ROTEM analysis in case of severe haemorrhagic shock (hybrid strategy).

In both group, platelets were given if the platelet number was < 50.10^9^ L^−1^, or 100.10^9^ L^−1^ in cases of haemorrhagic shock or severe brain injury [5]. RBC units were given to maintain haemoglobin above 7 g dL^−1^ [7].

MT was defined as the administration of at least 10 RBC units during the first 24 h of care. Patients were given tranexamic acid (1 g) during the first 3 h following the injury, either in the prehospital phase of care or at admission [5, 21]. After hospital admission, if necessary, a further 1 g was given in bolus or over an 8-h continuous infusion.

### Outcome measures

The primary clinical endpoint was the proportion of subjects who, at 24 h post-injury, were alive and free of MT [19]. Secondary endpoints included early death (< 24 h following admission), death at day 28, length of ICU stay (ICU LOS), MT rate, as well as the total amounts of BP (RBC, FFP, PC) and coagulation factors (FC and PCC).

### Cost analysis

We also compared the price of BP and coagulation factors among groups. Cost calculation was based on the following price: RBC (1 U: 179.7 €), FFP (1 U: 97.7 €), platelets (1 U: 82.1 €), PCC (1 U: 0.4 €), and fibrinogen (1 g: 499.3 €).

### Statistical analysis

Baseline characteristics were described by counts and percentages for categorical variables, medians, and interquartile ranges [IQR] for continuous variables. Normality of the distribution was tested using the Kolmogorov–Smirnov test. The Mann–Whitney U-test and Student t-test were used for continuous variables, as appropriate. Statistical differences between groups were evaluated using the Chi-squared or Fisher’s exact test, as appropriate. A 2-tailed *P* < 0.05 was considered significant. The number of missing data is presented in a Additional file 1: Table S1). For missing data (< 10% of missing data among the selected variables for the propensity score), we used multiple imputation by the chained equations (MICE). Then, the differences between groups were estimated using the paired T test for quantitative variables, and the Mc Nemar test for qualitative variables.

We described blood products transfused over 24 h to ISS score and the frequency of administration of FFP/PCC according to the ISS or admission lactate.

### Propensity score matching

A matching procedure was performed using propensity score and greedy matching for a 1:1 ratio. Variables included in the propensity score matching were selected a priori based on clinician expertise: sex, age, type of trauma (penetrating or blunt), systolic blood pressure (SBP), lactate and PT_ratio_ at admission, ISS and head abbreviated injury scale (AIS), Glasgow coma scale (GCS), and administration of tranexamic acid in the first 3 h following the injury. The caliper width was set at 0.1. To achieve an adequate balance among the variables, an interaction term was introduced between SBP and lactate at admission. The adequate balance among groups was assessed using the standardised mean deviation (SMD) of each variable of the propensity score. The balance among both groups was considered acceptable if the SMD was < 0.1 [29, 30].

Finally, the effect of the study group on the different endpoints was estimated by logistic regression. The odds ratio (OR) alongside their 95% confidence interval (95% CI) and *p* value were estimated. Statistical significance for the *p* value was set at 0.05.

Statistical analyses were performed using R software V 3.6.3 and the package mice and Matchlt [31, 32].

## Results

### Study population

**Fig. 2:**
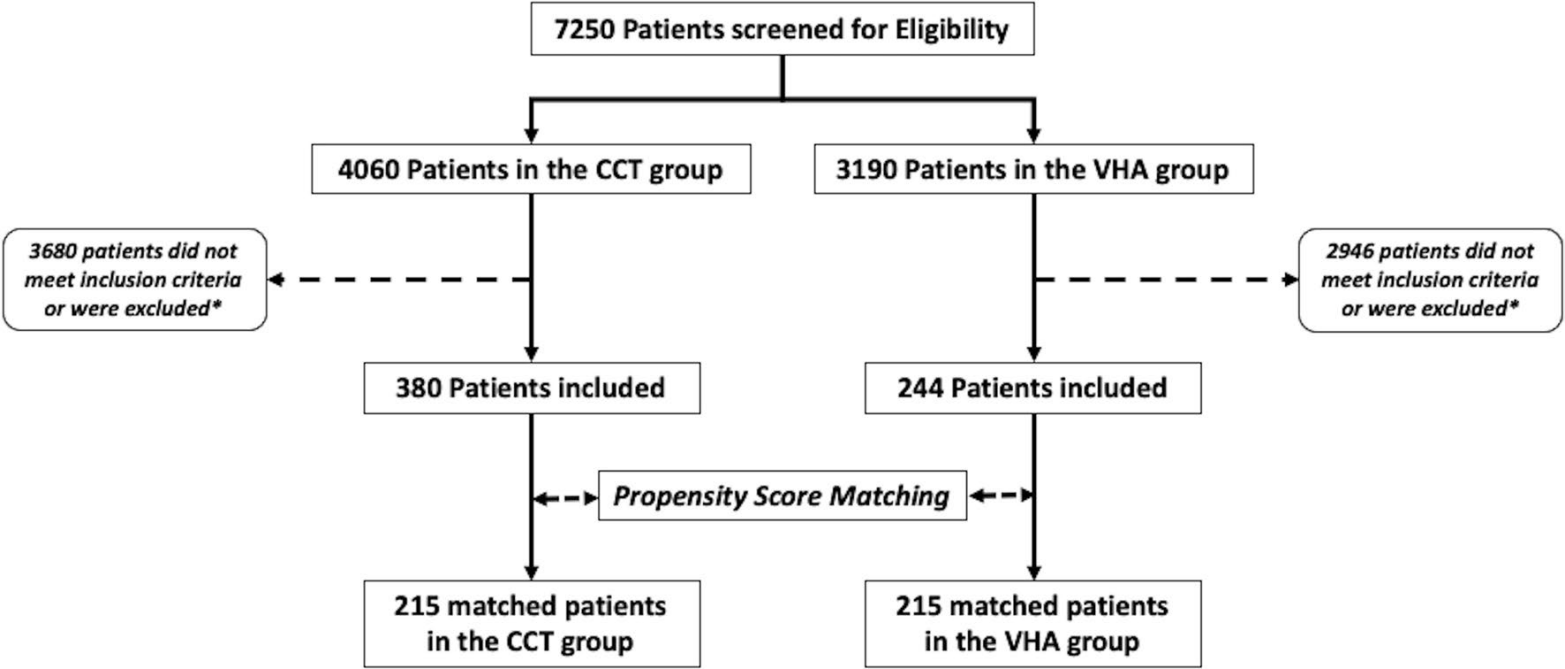
Study flowchart. *refers to exclusion criteria’s

From 1 January 2012 to 31 December 2019, 7250 patients were admitted to the 2 participating trauma centres. Among these, 624 patients were included in the study, 380 patients in the CCT group and 244 patients in the VHA group (Fig. 2). Patients in the CCT group were younger and suffered more frequently from penetrating injury (Table 1). Among patients suffering from penetrating trauma, the proportion of patients with gunshot wound was not different among groups (CCT group: 23 patients (32%) vs. VHA group: 13 patients (42%), *p*: 0.307).

**Table 1:** Demographics, admission characteristics, and blood products before and after matching

	Before matching		p value	After matching		p value
	CCT (*n* = 380)	VHA (*n* = 244)		CCT (*n* = 215)	VHA (*n* = 215)	
Age	35 [25–53]	43 [27–60]	** < *0.01***	38 [26–54]	40 [26–55]	0.53
Male sex	270 (71)	165 (68)	0.36	144 (66)	144 (67)	0.76
Penetrating Trauma	73 (19)	31 (13)	***0.03***	37 (17)	30 (14)	0.35
*Prehospital data*						
SBP—mmHg	101 [80–126]	99 [70–120]	0.08	100 [76–125]	98 [70–120]	0.20
Cardiac arrest	14 (4)	23 (9)	*** < 0.01***	8 (4)	21 (10)	***0.03***
GCS	13 [4–15]	13 [3–15]	0.21	11 [3–15]	13 [3–15]	0.90
Norepinephrine use	185 (49)	120 (49)	0.90	107 (50)	111 (51)	0.70
Fluids (ml)	1250 [750–1825]	1250 [750–2000]	0.18	1250 [750–1750]	1475 [888–2000]	***0.03***
*Admission data*						
SBP—mmHg	90 [69–120]	99 [77–119]	0.10	90 [68–120]	96 [75–115]	0.72
ISS	33 [22–43]	34 [24–45]	0.25	34 [22–45]	34 [22–45]	0.58
AIS head	2 [0–4]	3 [0–5]	***0.01***	3 [0–4]	2 [0–5]	0.96
Lactate—mmol/L	4.5 [2.8–7.4]	4.1 [2.3–7.1]	0.13	4.3 [2.6–7.4]	4.3 [2.4–7.5]	0.90
Haemoglobin—g/dL	9.5 [8.1–11.2]	9.6 [8.1–11.5]	0.38	9.5 [8.1–11.2]	9.7 [8.1–11.5]	0.49
TIC	299 (79)	157 (64)	** < *0.01***	170 (79)	168 (78)	0.81
PT_ratio_	1.5 [1.3–1.8]	1.4 [1.2–1.7]	** < *0.01***	1.5 [1.3–1.9]	1.4 [1.2–1.8]	0.10
Fibrinogen—g/L	1.5 [1.0–1.9]	1.5 [0.9–1.9]	0.63	1.4 [1.0–1.9]	1.5 [0.9–1.9]	0.17
Platelets—G/L	182 [129–231]	193 [142–240]	0.09	182 [135–221]	194 [145–240]	0.08
TXA H-3	301 (89)	237 (97)	** < 0.01**	207 (96)	208 (97)	0.79
*Blood products and coagulation factors*						
RBC—units	9 [6–14]	3 [2–6]	** < *0.01***	9 [6–12]	3 [2–6]	** < *0.01***
Massive transfusion	173 (46)	32 (13)	** < *0.01***	91 (42)	32 (15)	** < *0.01***
FFP—units	8 [4–13]	0 [0–3]	** < *0.01***	7 [4–12]	0 [0–4]	** < *0.01***
FFP or PCC given	368 (97)	104 (43)	** < *0.01***	209 (97)	94 (44)	** < *0.01***
PC—units	3.9 [0.0–7.8]	0 [0–0]	** < *0.01***	3.9 [0.0–7.8]	0 [0–0]	** < *0.01***
PC given	258 (68)	55 (23)	** < *0.01***	137 (64)	51 (24)	** < *0.01***
FC—g	3.0 [1.5–4.5]	3.0 [1.5–6.0]	** < *0.01***	3.0 [1.5–4.5]	3.0 [1.5–6.0]	0.06
FC given	302 (81)	187 (77)	0.25	167 (79)	167 (78)	0.71
Fib/RBC ratio	0.4 [0.3–0.5]	1.0 [0.8–1.5]	** < *0.01***	0.4 [0.3–0.5]	1.0 [0.8–1.5]	** < *0.01***
FFP/RBC ratio	0.8 [0.7–1.0]	0.7 [0.5–1.0]	0.04	0.8 [0.7–1.0]	0.7 [0.5–1.0]	0.08
*Outcomes*						
Alive and free of MT	185 (49)	186 (76)	** < *0.01***	112 (52)	162 (75)	** < *0.01***
Early death (< 24 h)	56 (15)	48 (20)	0.11	44 (20)	42 (20)	0.81
Survival at Day 28	272 (72)	151 (62)	***0.01***	144 (67)	137 (64)	0.48
ICU LOS (days)	9 [2–23]	7 [1–18]	** < *0.01***	8 [2–23]	7 [1–19]	0.19

Bolditalic value indicates *p* considered as significant if < 0.05

Data are *n* (%) or median [interquartile range]. *SBP* Systolic blood pressure, *GCS* Glasgow coma scale, *AIS* Abbreviated injury scale, *ISS* Injury severity score, *PT*_*ratio*_ Prothrombin time ratio, *TXA H-3* Tranexamic acid administration during the first 3 h following the injury. *RBC* Red blood cells, *MT* Massive transfusion, *FFP* Fresh frozen plasma, *PC* Platelets concentrate, *FC* Fibrinogen Concentrate, *PCC* Prothrombin complex concentrates

At the scene, at the first medical evaluation, there was no significant difference in vital signs but more patients in the VHA group experienced traumatic cardiac arrest (Table 1). There was also no significant difference in prehospital resuscitation (norepinephrine use and fluid infusion), but fewer patients in the CCT group received tranexamic acid within the first 3 h after being injured (Table 1).

On admission, systolic blood pressure was not different between groups (Table 1) and 6 patients were admitted in cardiac arrest (CCT group: 4 patients and VHA group: 2 patients; *p*: 0.771). AIS (head) was higher in the VHA group but ISS was not different between groups (Table 1). A TIC was observed more frequently in the CCT group than in the VHA group (Table 1). There was no other significant difference between groups in terms of laboratory findings (Table 1). Results of ROTEM analysis for the VHA patients before and after matching are shown in Additional file 1: Table S2.

**Fig. 3:**
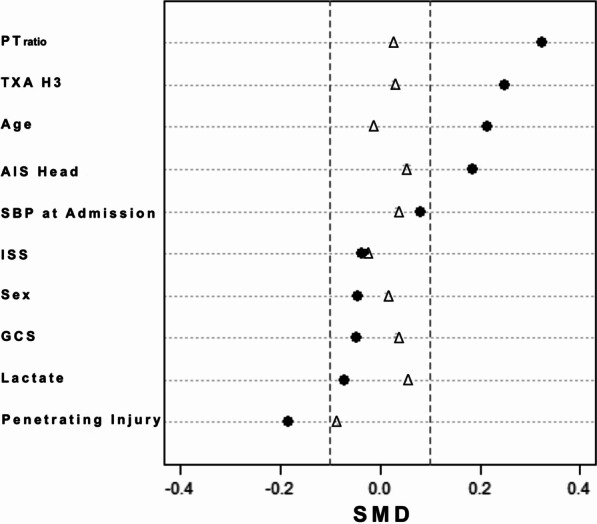
Standardised mean differences (SMD) before and after propensity score matching. SMD < 0.1 (discontinuous line) is often regarded as a good balance between groups. Mean differences before (✴) and after propensity score matching (△). TXA H-3: tranexamic acid administration during the first 3 h following the injury, *AIS* Abbreviated injury score, *SBP* Systolic blood pressure, *ISS* Injury severity score, *GCS* Glasgow coma scale, *PT*_*ratio*_ Prothrombin time ratio

After propensity score matching, 215 matched patients remained in each group for the final analysis. No significant difference was observed between study groups for the variables included in the score or for tranexamic acid administration (Table 1). The imbalance in patient characteristics before and after matching is presented in supplementary file (Fig. 3). However, we observed that patients in the VHA group experienced more frequently a cardiac arrest at the scene and received more fluids during prehospital care (Table 1).

### Outcomes

ICU LOS was higher in the CCT group as compared to the VHA group before matching. However, after matching, no significant difference was observed (Table 1).

Before matching and as compared to the CCT group, more patients in the VHA group were alive and free of MT at 24 h (Table 1 and 2). No significant difference in mortality was observed at 24 h but survival at day 28 was lower in the VHA group (Table 1 and 2).

**Table 2:** Primary outcome and mortality before and after propensity matching

Variable	Odds ratio (95% Confidence Interval)	*p* value
*Before matching (Ref: Group VHA)*		
Alive and free of massive transfusion	3.35 (2.36–4.79)	** < *0.01***
Massive transfusion	0.18 (0.12–0.27)	** < *0.01***
Early death (< 24 h)	1.42 (0.93–2.17)	0.11
Survival at day 28	0.64 (0.46–0.91)	***0.01***
*After matching (ref: Group VHA)*		
Alive and free of massive transfusion	2.81 (1.87–4.23)	** < *0.01***
Massive transfusion	0.24 (0.15–0.38)	** < *0.01***
Early death (< 24 h)	0.94 (0.59–1.51)	0.81
Survival at day 28	0.87 (0.58–1.29)	0.48

Bolditalic value indicates *p* considered as significant if < 0.05

After matching, the same result was observed with more patients in the VHA group alive and free of MT at 24 h as compared to the CCT group (Table 1 and 2). No significant difference in mortality was observed between groups at 24 h and at day 28 (Table 1 and 2). When the comparison was redone after removing patients with prehospital cardiac arrest, no significant difference was observed for early death (VHA group, OR (95% CI) 0.83 (0.49–1.42), *p*: 0.50) and survival at day 28 (VHA group, OR (95% CI) 0.98 (0.63–1.51), *p*: 0.91).

### Administration of blood products and coagulation factor concentrates

We observed an important reduction of MT before or even after propensity matching (Table 1). Before matching, patients in the VHA group received significantly fewer BP than those in the CCT group (Table 1). They also received less frequently FFP/PCC or platelets and had a lower FFP/RBC ratio. In the VHA group, patients received more fibrinogen and had a higher Fib/RBC ratio (Table 1). Forty-four (18%) patients in the VHA group received PCC (median [IQR]: 2000 IU [1500–2000]).

**Fig. 4:**
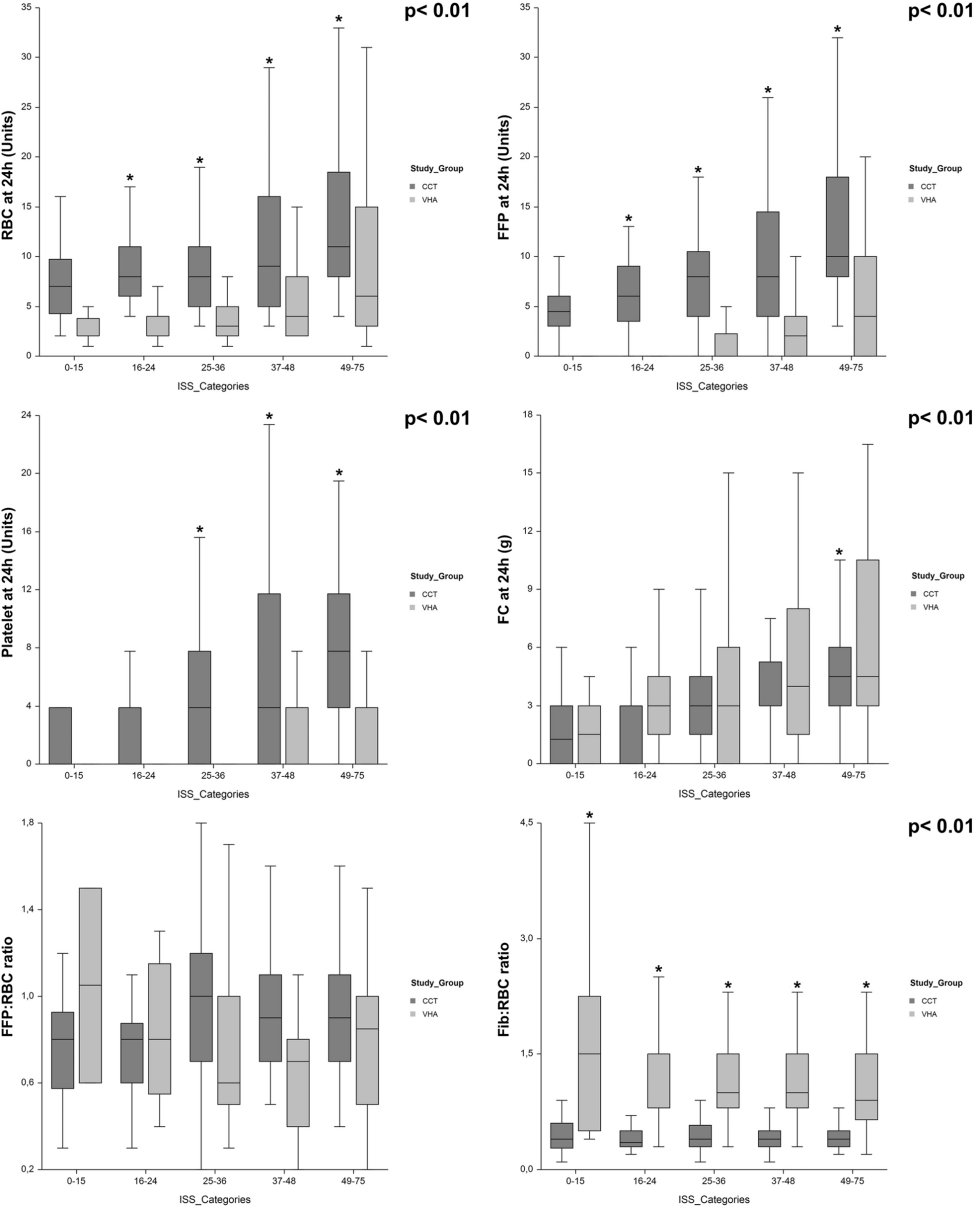
Box plot for the comparison of blood products (RBC: red blood cell, FFP: fresh frozen plasma) and fibrinogen concentrate (FC) administration according to injury severity in the matched cohort. *p* value refers to between group comparison whereas *p < 0.01 refers to the comparison for each ISS category. Comparison made using ANOVA and the Tukey–Kramer multiple comparison test. Data are median [interquartile range] and whisker boundaries corresponding to box edge ± 1.5 IQR

**Fig. 5:**
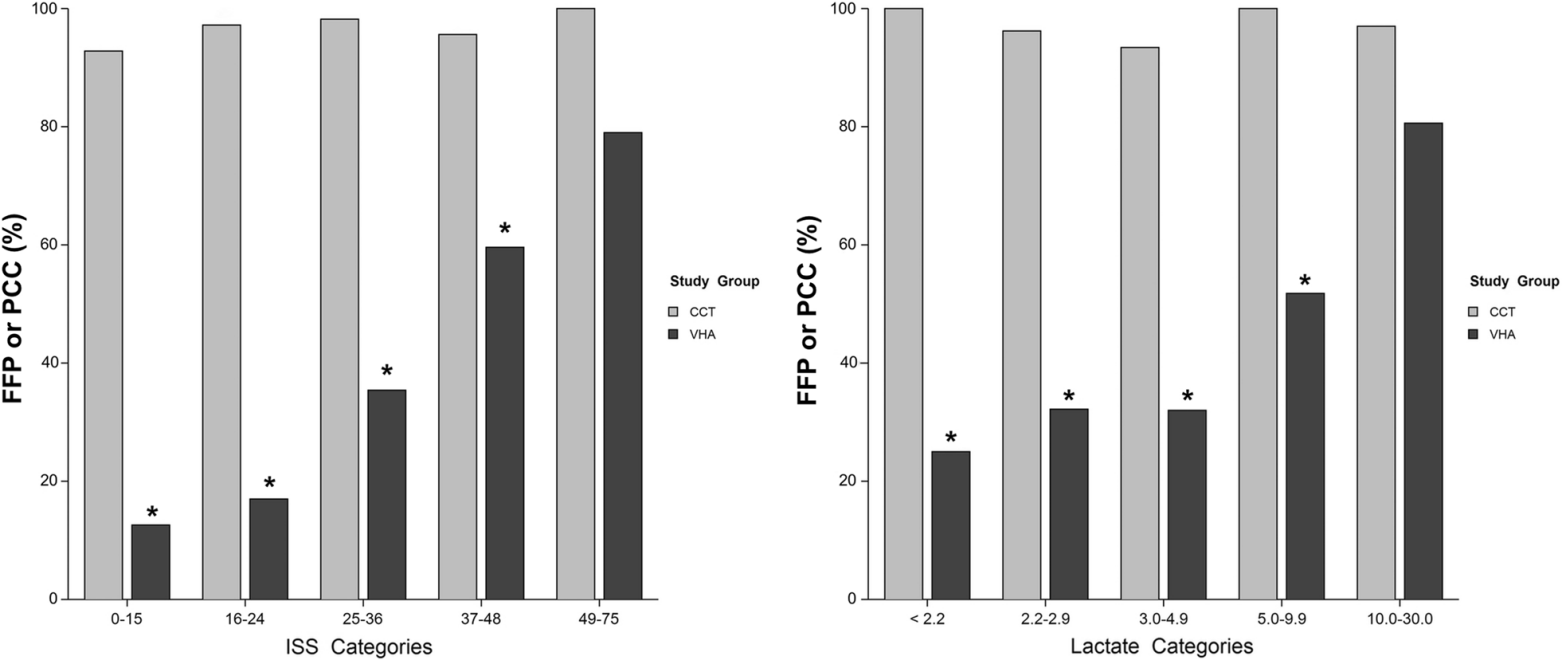
Comparison of the administration rate of FFP or PCC according to injury severity or admission lactate (mmol L^−1^) in the matched cohort. *p < 0.05 for the comparison between ISS or lactate categories. Comparison made using ANOVA and the Tukey–Kramer multiple comparison test. Data are mean %

After matching, patients in the VHA group received significantly fewer BP at 24 h and had a higher Fib/RBC ratio (Table 1). As observed previously, patients in the VHA group received less frequently FFP and platelets and when FFP and platelet were given, they received a lower amount **(**Table 1**)**. Thirty-six patients (16%) patients in the VHA group received PCC (median [IQR]: 2000 IU [1500–2750]). When the comparison was done according to the ISS, patients in the VHA group received fewer BP (RBC, FFP, platelets), and more fibrinogen **(**Fig. 4). When we compared the frequency of administration of FFP/PCC according to the ISS or admission lactate, we observed that the lower the injury severity or lactate the greater was the difference between study groups (Fig. 5). For example, patients in the CCT group with an ISS 0–15 were nine times more likely to receive FFPs; for the patients who were the most severely injured (ISS > 48 or Lactate > 9.9 mmol.L^−1^), no significant difference was observed.

### Haemorrhage control procedures

In the matched cohort, we observed that the number of patients undergoing a haemostatic procedure was not significantly different in both study groups (CCT: 101 patients (47%) vs. VHA: 83 patients (39%), *p*: 0.08). More patients in the CCT group required interventional radiology and more patients in the VHA group did not have a haemorrhage control procedure (Table 3). As well, there was no significant difference in the delay between admission to the start of procedure (CCT: 90 min [53–120] vs. 75 min [22–127], *p*: 0.06).

**Table 3:** Bleeding control procedures in the 2 study groups

	ALL	CCT	VHA
Laparotomy/pelvic packing	96 (22)	47 (22)	49 (23)
Thoracotomy	25 (6)	11 (5)	14 (7)
Interventional radiology	63 (15)	43 (20)	20 (9)
No procedure	246 (57)	114 (53)	132 (61)

Date are *n* (%). Difference between group was assessed with Pearson’s Chi-square test (*p*: 0.0176)

### Comparison to the cost of blood products and coagulation factor concentrates

In the unmatched cohort, the overall cost including BP and coagulation factor concentrates was greater in the CCT group (median [IQR]: 4104 € [2687–6372]) than in the VHA group (2347 € [1108–4626]), *p* < 0.001). When the comparison was performed on the matched cohort, this difference remained (CCT, median [IQR]: 4092 € [2510–5916] vs. VHA, 2357 € [1108–5020], *p* < 0.001). Costs of BP and coagulation factors before and after matching are detailed in Additional file 1: Table S3.

## Discussion

In the present study, we observed in an injured population with a high probability of TIC that patients treated according to a VHA-based algorithm were more frequently alive and free of MT than patients treated according to a CCT-based algorithm. However, this did not translate into an improvement of the mortality at 24 h or on day 28. We also observed that there was a strong reduction in the use of BP that was associated with an important decrease in the cost of blood resuscitation, including coagulation factors.

These results are consistent with several other studies on viscoelastic techniques that found a decrease in the administration of RBCs, FFPs, and PCs [17, 18, 33]. However, it has been argued that the reduction in BP use observed in these studies was perhaps not solely related to VHA goal-directed management strategies but rather to the implementation of a bundle of care including VHA management, damage control strategies, and tranexamic acid administration [17, 18]. This study provides an answer to these criticisms because here the two groups studied benefited from the administration of tranexamic acid and Damage Control strategy. Recently, a recent randomised controlled study comparing VHA-based algorithms (TEG or ROTEM) to an algorithm based on CCT, failed to find a difference in BP administration, MT rate, or even in mortality [19]. The main criticism of this study was that the patients included were not sufficiently severely injured thus decreasing the likelihood of demonstrating a difference between the study groups [20]. To address this limitation, we decided to include only patients who had received at least one RBC, to have increased severity injury and higher probability of TIC, as illustrated herein by a higher ISS (median ISS 33 vs. 26) and a higher number of patients with TIC (> 75% vs. 25%) as compared to the ITACTIC cohort. A decrease in mortality might have been expected because it has been previously shown that the use of VHAs is associated with a reduction to the time for diagnosis and treatment of TIC [27, 34]; more importantly, the large reduction in BP use might have reduced the occurrence of complications in the ICU, such as TRALI, infections, MOF, and thrombotic events as several studies have reported a direct relationship between BP administration and the occurrence of these, or even an increase in mortality [30, 35–37]. However, these complications were not recorded in the trauma registries used herein, and the sample was probably too small to expect to find a difference in prognosis.

We observed that the difference in BP administration between the 2 groups was greater for the lowest ISS categories, suggesting that the effects of the VHA were greatest among these patients of intermediate severity but with a high probability of coagulopathy. The difference in BP administration was smaller for the most severe patients because, for these patients, the urgency of the situation often led to transfusing them first and then adapting the treatment with VHA (hybrid strategy). It should also be noted that a benefit of a high ratio of BP has only been demonstrated in the case of uncontrolled haemorrhagic shock and MT and outside of this situation of MT, no benefit has been demonstrated [38]. To avoid over-reacting and administering BP unnecessarily, as the clinical diagnosis of TIC at admission is difficult, the results presented herein underline the value of VHAs to guide the administration of BP in clinical situations under control and without a high probability of MT.

With the more rapid and targeted treatment of coagulopathy, using a VHA-based algorithm, we would have expected to observe not only a decrease in the administration of blood products but also a decrease in the number of bleeding control procedures (laparotomy, thoracotomy or interventional radiology procedure). However, and probably due to the small number of patients in each group, we did not observe a significant decrease in the number of procedures needed to control bleeding in the VHA group, even though fewer patients required an interventional radiology procedure. This is an interesting point and will require further investigation in a larger cohort of patients. It should also be noted that in cardiac surgery the use of VET was not associated with a reduction in the number of reoperation for bleeding [39].

Another finding was the strong reduction in the cost of BP, despite the increased administration of fibrinogen and PCCs in the VHA group. This cost reduction was approximately 40% per patient. Such a reduction to the cost has been previously observed in trauma and cardiac surgery [16, 17, 40]. This is important information, but consideration should also be given to the overall cost, including the purchase of viscoelastic analysers and their depreciation, maintenance and reagent prices. The cost of a ROTEM analysis (2 channels) was estimated at 30.1 euros compared to 2.1 euros for standard tests [41]. From a previous work, we observed that for patients receiving RBC, an average of 2.3 ± 1.4 ROTEM analyses were performed [42]. Given that the average cost difference in the matched cohort was 836.8 ± 408 euros and considering the cost of the ROTEM analysis mentioned above, the difference remains largely positive and the saving per patient significant.

### Clinical implication

Early identification of TIC is often a challenge for clinicians and VHA can help them to quickly give the right product to the right patient and then, to avoid unnecessary administration of BP, which is important in situations of BP shortage. This concept of identifying *a haemostatic phenotype defined by VHA to guide BP and coagulation factors administration is part of precision-based medicine (PBM) and is gaining importance in in many clinical situations, such as trauma.*

### Strengths and limitations

Our study has important strengths. The effects observed in this study were not the result of the implementation of a bundle of care, as previously observed, but the results of the use of a VHA-based algorithm. DCR strategies and TXA administration being commonly used in both study centre. The second strength of that study was the possibility to have most of the patients in each group with a TIC (> 75%). This category of patients being the most severely injured and the most difficult to capture in prospective study. It is probably for these patients, with a TIC, that viscoelastic techniques are the most interesting and provide the most clinical benefit.

Our study also has limitations. First, the retrospective nature of the study design leaves the possibility of residual confounding. For example, the trauma networks of the 2 study centres are organised differently, an inclusive model for the VHA centre and an exclusive model for the CCT centre. This may have affected the results since Utter et al. suggested that the prognosis of patients could be better in a highly exclusive system [43]. However, in a recently published study, it was found that transport times were shorter in a region with an inclusive organisation as compared to a region with an exclusive organisation [44]. In the present study, it is possible that patients in the CCT group arrived later to the hospital after being injured than those in the VHA group, but prehospital time was not recorded for patients in the latter. Hence, before matching, patients in the CCT group had higher PT_ratio_ at admission, after receiving similar prehospital resuscitation but after matching, the difference was no longer significant. Second, the use of propensity score matching reduced the number of patients, reducing the power of the study and possibly explaining the lack of benefit on overall survival. Third, the inclusion of patients who received at least 1 RBC unit within 24 h attesting a significant bleeding may have contributed to an underestimation of the results of the present study. Indeed, during the study period, 92 patients in the VHA group (median ISS: 29) had a TIC and received fibrinogen concentrate without RBC transfusion. They may have had their coagulopathy corrected rapidly with less bleeding and thus no RBC transfusion. It would therefore be of interest to investigate in a future study, patients with a high probability of having a TIC, having been or not transfused. Some patients received a prehospital transfusion. For example, in the VHA group, 40 patients received a mean of 1.9 ± 0.9 units of RBC (no plasma). In the CCT group, only 8 patients received RBCs (4 patients, 1.8 ± 0.8 units) and/or plasma (4 patients, 1 unit), but data were only available for 180 patients. It remains unclear how this may have influenced the results, but it is conceivable that this prehospital administration of RBCs, particularly in the VHA group, may have contributed to minimizing the difference between the 2 groups. Fourth, patients in the VHA group who received less blood products received approximately 4 times less fluid volume. However, it is not possible to know whether the final volume of fluid administered (blood products plus crystalloid or colloid) in the first 24 h was similar in the 2 groups as this information was not recorded. It is possible that all or part of the observed BP volume difference between the 2 groups was compensated in the VHA group by crystalloid or colloid. This should be considered in future studies because of the presumed link between fluid volume administered and the occurrence of multiple organ failure or ARDS [45]. Finally, we did not take into account the time bias and practices may have changed over time in the two centres with the implementation at different times of recent concepts (increase in platelet/RBC ratio, use of REBOA…).

## Conclusion

In the present study, using a VHA-based transfusion strategy was associated with an increased probability of being alive and free of MT without any difference in mortality at 24 h as well as at day 28. A VHA-based strategy was associated with an important decrease in the use of BP and their related cost. These promising results will have to be confirmed in the future in a randomised study, including a majority of patients with a TIC.

## Supplementary Information

**Additional file 1: Table S1.** Missing data. **Table S2.** ROTEM analysis for patients in the VHA group at admission. **Table S3.** Blood products and coagulation factors cost before and after matching in euros

## Data Availability

On demand to the authors.
