# Supplementary material for: Thromboelastometry-guided haemostatic resuscitation in severely injured patients: a propensity score-matched study
Source: Crit Care. 2023 Apr 13;27:141. doi: 10.1186/s13054-023-04421-w (PMC10103518; doi:10.1186/s13054-023-04421-w)
Supplement: Supplementary file 1 — Additional file 1: Table S1. Missing data. Table S2. ROTEM analysis for patients in the VHA group at admission. Table S3. Blood products and coagulation factors cost before and after matching in euros [file 13054_2023_4421_MOESM1_ESM.docx]

**Supplementary File.**

**Table s1.** Missing data.

|  | **Before matching** | | **After matching** | |
| --- | --- | --- | --- | --- |
|  | **CCT (n = 380)** | **VHA (n = 244)** | **CCT (n = 215)** | **VHA (n = 215)** |
| Age | 0 (0) | 0 (0) | 0 (0) | 0 (0) |
| Male sex | 0 (0) | 0 (0) | 0 (0) | 0 (0) |
| Penetrating / Blunt Trauma | 1 (<1) | 0 (0) | 0 (0) | 0 (0) |
|  |  |  |  |  |
| ***Prehospital Data*** |  |  |  |  |
| SBP - mmHg | 140 (37) | 19 (8) | 80 (37) | 16 (7) |
| GCS | 2 (<1) | 0 (0) | 0 (0) | 0 (0) |
| Norepinephrine use | 0 (0) | 0 (0) | 0 (0) | 0 (0) |
| Fluids (ml) | 10 (3) | 12 (5) | 7 (3) | 11 (5) |
|  |  |  |  |  |
| ***Admission Data*** |  |  |  |  |
| SBP - mmHg | 0 (0) | 0 (0) | 0 (0) | 0 (0) |
| ISS | 0 (0) | 0 (0) | 0 (0) | 0 (0) |
| Lactate – mmol/L | 6 (2) | 50 (20) | 0 (0) | 0 (0) |
| Haemoglobin – g/dL | 4 (1) | 3 (1) | 3 (1) | 3 (1) |
| PT_ratio_ | 0 (0) | 29 (12) | 0 (0) | 0 (0) |
| Fibrinogen – g/L | 8 (2) | 29 (12) | 10 (5) | 26 (12) |
| Platelets – G/L | 6 (2) | 6 (2) | 5 (2) | 6 (3) |
| TXA received < 3h | 41 (11) | 0 (0) | 0 (0) | 0 (0) |
|  |  |  |  |  |
| **Blood products at 24h** |  |  |  |  |
| RBC - units | 0 (0) | 0 (0) | 0 (0) | 0 (0) |
| FFP – units | 0 (0) | 0 (0) | 0 (0) | 0 (0) |
| PC - units | 1 (<1) | 0 (0) | 0 (0) | 0 (0) |
| FC - g | 5 (1) | 0 (0) | 4 (2) | 0 (0) |
|  |  |  |  |  |
| **Outcomes** |  |  |  |  |
| ICU LOS (days) | 0 (0) | 0 (0) | 0 (0) | 0 (0) |
| Early Death (< 24h) | 0 (0) | 0 (0) | 0 (0) | 0 (0) |
| Survival (Day 28) | 0 (0) | 0 (0) | 0 (0) | 0 (0) |

Data are n (%).

**Table s2.** ROTEM analysis for patients in the VHA group at admission.

|  | **Before Matching** | **After Matching** |
| --- | --- | --- |
| **N** | 226 | 202 |
| **EXTEM CT (s)** | 78 [63–117] | 79 [63–117] |
| **EXTEM A5 (mm)** | 34 [24–41] | 34 [24–41] |
| **EXTEM MCF (mm)** | 55 [47–61] | 55 [47–61] |
| **EXTEM ML (%)** | 3 [1–6] | 3 [1–5] |
| **FIBTEM A5 (mm)** | 6 [3–9] | 6 [3–9] |
| **FIBTEM MCF (mm)** | 8 [4–11] | 7 [4–11] |

Data are median and interquartile range. CT: clotting time; A5: clot amplitude at 5 min; MCF: maximum clot firmness.

In the VHA group, 18 (before matching) and 13 (after matching) patients did not have ROTEM analysis at admission but subsequently did so during care.

**Table s3.** Blood products and coagulation factors cost before and after matching in euros.

|  | **Before matching** | | **After matching** | |
| --- | --- | --- | --- | --- |
|  | **CCT (n = 380)** | **VHA (n = 244)** | **CCT (n = 215)** | **VHA (n = 215)** |
| **RBC** | 1617 [1078-2515] | 539 [359-1078] | 1617 [1078-2156] | 539 [359-1078] |
| **FFP** | 778 [389-1241] | 0 [0-292] | 684 [391-1172] | 0 [0-391] |
| **PC** | 320 [0-640] | 0 [0-0] | 320 [0-640] | 0 [0-0] |
| **All BP** | 2686 [1705-4227] | 665 [359-1353] | 2579 [1594-3911] | 719 [359-1438] |
| **FC** | 1498 [749-2246] | 1498 [749-2996] | 1498 [749-2247] | 1498 [749-2996] |
| **PCC** | 0 [0-0] | 0 [0-0] | 0 [0-0] | 0 [0-0] |

Data median [interquartile range].
